# Mutual Associations of Exposure to Ambient Air Pollutants in the First 1000 Days of Life With Asthma/Wheezing in Children: Prospective Cohort Study in Guangzhou, China

**DOI:** 10.2196/52456

**Published:** 2024-04-17

**Authors:** Fenglin Tian, Xinqi Zhong, Yufeng Ye, Xiaohan Liu, Guanhao He, Cuiling Wu, Zhiqing Chen, Qijiong Zhu, Siwen Yu, Jingjie Fan, Huan Yao, Wenjun Ma, Xiaomei Dong, Tao Liu

**Affiliations:** 1 Department of Public Health and Preventive Medicine School of Medicine, Jinan University Guangzhou China; 2 China Greater Bay Area Research Center of Environmental Health School of Medicine, Jinan University Guangzhou China; 3 Department of Neonatology, The Third Affiliated Hospital of Guangzhou Medical University Guangzhou China; 4 Guangzhou Panyu Central Hospital Guangzhou China; 5 Department of Prevention and Health Care, Shenzhen Maternity and Child Healthcare Hospital, Southern Medical University Shenzhen China

**Keywords:** pregnancy, air pollution, asthma, wheezing, birth cohort, children

## Abstract

**Background:**

The first 1000 days of life, encompassing pregnancy and the first 2 years after birth, represent a critical period for human health development. Despite this significance, there has been limited research into the associations between mixed exposure to air pollutants during this period and the development of asthma/wheezing in children. Furthermore, the finer sensitivity window of exposure during this crucial developmental phase remains unclear.

**Objective:**

This study aims to assess the relationships between prenatal and postnatal exposures to various ambient air pollutants (particulate matter 2.5 [PM_2.5_], carbon monoxide [CO], sulfur dioxide [SO_2_], nitrogen dioxide [NO_2_], and ozone [O_3_]) and the incidence of childhood asthma/wheezing. In addition, we aimed to pinpoint the potential sensitivity window during which air pollution exerts its effects.

**Methods:**

We conducted a prospective birth cohort study wherein pregnant women were recruited during early pregnancy and followed up along with their children. Information regarding maternal and child characteristics was collected through questionnaires during each round of investigation. Diagnosis of asthma/wheezing was obtained from children’s medical records. In addition, maternal and child exposures to air pollutants (PM_2.5_ CO, SO_2_, NO_2_, and O_3_) were evaluated using a spatiotemporal land use regression model. To estimate the mutual associations of exposure to mixed air pollutants with the risk of asthma/wheezing in children, we used the quantile g-computation model.

**Results:**

In our study cohort of 3725 children, 392 (10.52%) were diagnosed with asthma/wheezing. After the follow-up period, the mean age of the children was 3.2 (SD 0.8) years, and a total of 14,982 person-years were successfully followed up for all study participants. We found that each quartile increase in exposure to mixed air pollutants (PM_2.5_, CO, SO_2_, NO_2_, and O_3_) during the second trimester of pregnancy was associated with an adjusted hazard ratio (HR) of 1.24 (95% CI 1.04-1.47). Notably, CO made the largest positive contribution (64.28%) to the mutual effect. After categorizing the exposure according to the embryonic respiratory development stages, we observed that each additional quartile of mixed exposure to air pollutants during the pseudoglandular and canalicular stages was associated with HRs of 1.24 (95% CI 1.03-1.51) and 1.23 (95% CI 1.01-1.51), respectively. Moreover, for the first year and first 2 years after birth, each quartile increment of exposure to mixed air pollutants was associated with HRs of 1.65 (95% CI 1.30-2.10) and 2.53 (95% CI 2.16-2.97), respectively. Notably, SO_2_ made the largest positive contribution in both phases, accounting for 50.30% and 74.70% of the association, respectively.

**Conclusions:**

Exposure to elevated levels of mixed air pollutants during the first 1000 days of life appears to elevate the risk of childhood asthma/wheezing. Specifically, the second trimester, especially during the pseudoglandular and canalicular stages, and the initial 2 years after birth emerge as crucial susceptibility windows.

**Trial Registration:**

Chinese Clinical Trial Registry ChiCTR-ROC-17013496; https://tinyurl.com/2ctufw8n

## Introduction

Asthma is one of the major respiratory diseases. It is also the most common noncommunicable disease in children and imposes a huge economic and disease burden worldwide [1]. Asthma is estimated to affect around 14% of children worldwide, with its prevalence reported to be on the rise [2]. It is well-known that childhood asthma increases the burden on society in terms of disruption to children’s lives, reduced physical ability, increased caregiver strain, and various direct medical costs. Wheezing during early childhood is frequently regarded as the primary symptom linked to asthma later in life [3]. Research has shown that the intricate interplay of environmental exposures and genetic susceptibility can contribute to the onset of wheezing and asthma [4].

The first 1000 days of life, encompassing pregnancy and the initial 2 years after birth, represent a crucial period for human health development and interventions [5]. Lung and airway development commences between the 4th and 7th weeks of pregnancy, reaching the alveolar stage by 36 weeks. During pregnancy, fetal cells exhibit more rapid replication and differentiation compared with mature cells, making them highly responsive to external signals [6]. Consequently, they are particularly susceptible to external exposure events. Air pollution, for instance, can disrupt alveolarization, leading to compromised lung development and function postnatally [7,8]. The lungs undergo growth from conception through early adulthood, with the prenatal and early postnatal phases being particularly critical [9]. There is evidence suggesting that exposure to air pollution during early life can trigger the onset of asthma or wheezing and exacerbate preexisting conditions [5,10,11]. A review, which has consolidated the link between exposure to air pollutants throughout the first 1000 days of life and the occurrence of asthma or wheezing in childhood, concluded that exposure to particulate matter (PM_x_) and nitrogen oxide (NO_x_) during pregnancy and the initial 2 years of life correlated with a heightened risk of developing asthma. Notably, the second trimester of pregnancy emerged as a particularly significant period [5]. In addition, numerous studies have established connections between exposure to other pollutants such as ozone (O_3_), carbon monoxide (CO), and sulfur dioxide (SO_2_) during pregnancy or childhood and the onset of asthma in children [12,13]. For instance, Lin et al [12] investigated the correlation between long-term exposure to ambient O_3_ and hospital admissions for asthma in children. They discovered a positive association between asthma admissions and increased levels of ambient O_3_ exposure. However, many of these studies solely explored the connection between individual pollutants and childhood asthma, without accounting for the combined impact of outdoor pollutants.

Outdoor air pollution typically arises as a blend of various pollutants, yet many previous studies in both humans and animals have concentrated on the influence of individual pollutants on asthma or wheezing [14,15]. This narrow focus often obscures the comprehensive health implications of the pollutant mixture. Recognizing that air pollution comprises a combination of PM and gaseous pollutants, whose collective impact on health may diverge from that of individual pollutants, we characterized the collective influence of 5 pollutants (PM_2.5_, SO_2_, nitrogen dioxide [NO_2_], CO, and O_3_) on childhood asthma development as their mutual association. Guarnieri and Balmes [16] have underscored the correlation between traffic-related air mixture pollutants (TRAPs) and asthma exacerbation, with a particular emphasis on the effects of TRAP as a mixture. Their review concluded that exposure to TRAP mixtures was not only accountable for triggering new asthma attacks but also for exacerbating existing asthma conditions. Research that examines multiple air pollutants collectively can contribute to a thorough and structured comprehension of the health hazards posed by air pollutants on asthma occurrence. Such studies offer a scientific foundation for potential public health interventions. With the ongoing global climate change, the composition of air pollutants is anticipated to become increasingly intricate. Consequently, estimating the combined impacts of air pollutants on asthma incidence is crucial for implementing early prevention measures against asthma and allergies in children.

In this study, we undertook a prospective birth cohort investigation into the prenatal environment and offspring health in Guangzhou, China. Our objective was to assess the relationships between prenatal and postnatal exposures to various ambient air pollutants (PM_2.5_, CO, SO_2_, NO_2_, and O_3_) and the onset of childhood asthma or wheezing. Furthermore, we aimed to pinpoint the potential sensitivity window for the effects of air pollution.

## Methods

### Study Settings and Participants

All participant data were sourced from the Prenatal Environments and Offspring Health (PEOH) cohort study conducted at Panyu Central Hospital in Guangzhou, China. Detailed descriptions of this cohort study have been provided in previous publications [15,17,18]. Further information regarding the population within the hospital’s catchment area, along with some basic demographic details concerning the broader populations of Guangzhou and China, can be found in Multimedia Appendix 1. Pregnant women were enrolled in the study at the antenatal care unit, adhering to the following inclusion criteria: (1) gestational age <13 weeks; (2) between 18 and 50 years of age; and (3) absence of significant medical conditions, including hyperthyroidism, hypertension, chronic kidney disease, tuberculosis, and mental illness. All eligible women underwent face-to-face interviews to collect baseline information, and they were subsequently followed up during hospital delivery. Furthermore, we administered follow-up surveys on the children during their hospital visits. Throughout the follow-up period, we excluded cases involving multiple pregnancies and study participants with missing key variables. Recruitment for our study commenced in January 2016 and continued until July 31, 2020, when the follow-up concluded.

### Baseline Investigation

The baseline survey for the study was initiated in January 2016 and concluded in December 2017. A total of 4928 pregnant women were successfully recruited and their personal profiles were established. The collected information encompassed their demographic characteristics, lifestyle behaviors, changes in home address, living environment at home, work environment, activity patterns, medical history, diet during pregnancy, and antenatal care records.

### Follow-Up Investigation

**Figure 1:**
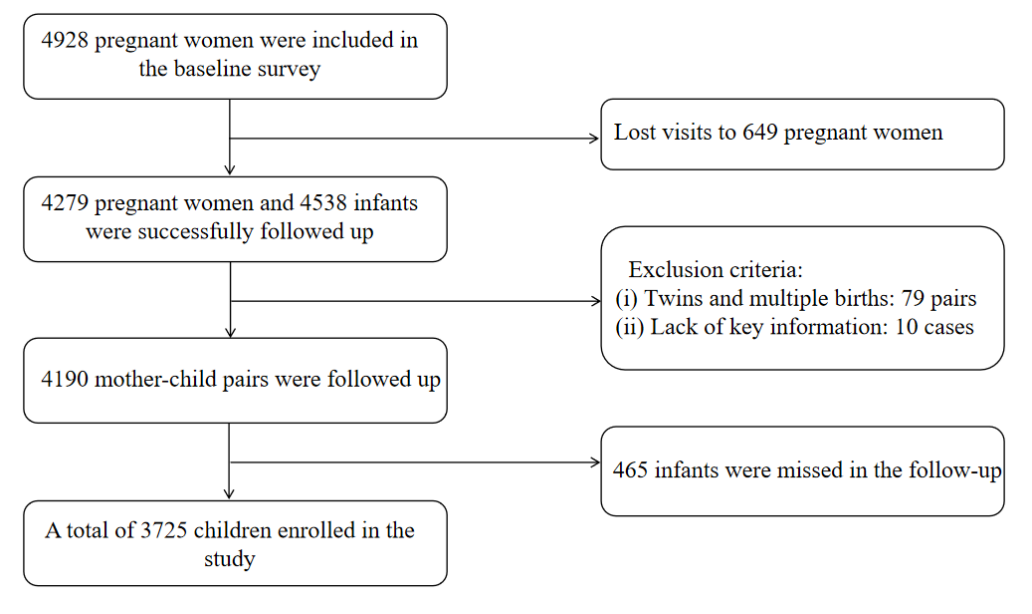
Flow chart of the progress of the research topic.

As illustrated in Figure 1, of the 4928 pregnant women enrolled in the baseline survey, 4279 were effectively followed up during their hospitalization for delivery. Throughout this period, maternal information was gathered via a follow-up questionnaire, while neonatal birth records were extracted from maternal medical records. Following the exclusion of cases involving multiple births (n=79) and those with missing key variables (n=10), a total of 4190 mother-child pairs were included in the follow-up. All children who attended child health clinics, pediatric outpatient clinics, or emergency departments were tracked for follow-up. Clinical symptoms, diagnostic disorders, and anthropometric data were extracted from their medical records, and questionnaires were administered to their parents or accompanying individuals. Throughout the follow-up process, a total of 465 infants were not successfully followed up. The final follow-up survey was concluded by July 31, 2020, with a total of 3725 children enrolled in the study (Figure 1).

### Major Health Outcomes

The health outcomes in this study were delineated in accordance with the 10th edition of the International Classification of Diseases (ICD-10), encompassing asthma (J45) and wheezing (R06). Asthma was characterized by recurrent and variable clinical manifestations, such as wheezing, chest tightness, persistent cough, shortness of breath, and restricted expiratory airflow. Our health outcomes also incorporated wheezing because of the challenges in diagnosing early childhood asthma, which led to a relatively low number of children diagnosed with asthma (n=17). Conversely, early childhood wheezing is frequently regarded as the primary symptom linked to asthma in later life and holds significant prognostic value for the early detection of asthma. Consequently, we ultimately delineated the health outcome as the first admission to the hospital for asthma or wheezing, using the time of initial occurrence of asthma/wheezing as the onset time. For cases where asthma or wheezing did not manifest, the follow-up continued until July 31, 2020. Eventually, to enhance statistical power, we combined asthma and wheezing (n=392) as a unified outcome.

### Ethical Considerations

The PEOH study received approval from the Ethics Committee of the Guangdong Center for Disease Control and Prevention under the reference number W96027E-2015001. Furthermore, it was registered in the Chinese Clinical Trial Registry with the registration number ChiCTR-ROC-17013496. This analysis was approved by the Ethics Committee of the Faculty of Medicine of Jinan University under the approval number JNUKY-2023-0075. During the establishment of the original cohort, comprehensive details about the study were provided to the recruited women, and informed consent was obtained from them through signed documents under conditions of autonomy. When accessing the data set for this research, it was entirely deidentified, and there was no feasible way to link the data to the women, either through a key in the coding system or otherwise. Stringent measures have been implemented to safeguard the personal information of the women.

### Exposure Assessment of Air Pollution

In this study, we incorporated 5 major ambient air pollutants (PM_2.5_, SO_2_, NO_2_, CO, and O_3_). To evaluate each individual’s weekly exposure to these air pollutants, we used a spatiotemporal land use regression (ST-LUR) model, which has been extensively described in our team’s previous publications [18-20]. In particular, we collected spatiotemporal data from air quality monitoring stations located throughout Guangdong Province to establish the ST-LUR model. We used an inverse distance weighting method to extract the weekly visibility of each address. This method relied on the latitude and longitude information to calculate the proximity between each participant’s home and work addresses and the air quality monitoring stations. Population density, road length, and land use–type data were obtained for each air quality monitoring station address within a radius of 1300 m. Predictor variables for both residential and work addresses of each participant were incorporated into the model. Subsequently, we used these variables to predict the weekly average concentrations of air pollutants at each address throughout the pregnancy period. To evaluate mothers’ exposure to air pollutants during pregnancy, we forecasted weekly pollutant concentrations at their individual residences and workplaces. Subsequently, we multiplied these concentrations by the percentage of time spent outdoors and indoors, respectively, to estimate individual daily time-weighted values of pollutant exposure. These daily time-weighted estimates were aggregated to calculate mean pollutant exposures for the entire gestation period, as well as for specific trimesters: the first trimester (1-13 gestational weeks [GWs]), the second trimester (14-27 GWs), and the third trimester (≥28 GWs).

Moreover, pinpointing a susceptible exposure window will enhance antenatal care and enable clinician interventions to mitigate the onset of asthma. The majority of studies investigating the relationship between air pollutants and asthma have traditionally used standard exposure window assignments, typically defined clinically as 3-month periods: the first trimester, the second trimester, and the third trimester. Indeed, such a division fails to consider the intricacies of respiratory system development and may not accurately elucidate the relationship. Consequently, inconsistencies in determining the window of susceptibility to air pollution have been observed in previous studies [21]. Thus, this study segmented the entire pregnancy period into distinct developmental stages of the respiratory system: the embryonic stage (0-6 GWs), pseudoglandular stage (6-16 GWs), canalicular stage (16-24 GWs), saccular stage (24-36 GWs), and alveolar stage (>36 GWs), drawing from existing literature on fetal respiratory system development [22-24].

Given that children primarily spend a significant portion of their early life at or near their homes, our postnatal exposure assessment initially involved estimating daily pollutant concentrations at the home address for children aged 0-2 years. To identify sensitive exposure time windows during childhood, considering the considerable changes in children’s activity patterns as they grow, we calculated air pollutant concentrations for 2 distinct periods: from birth to 12 months (the first year of life) and from birth to 24 months (the first 2 years of life) [25].

### Covariates

Upon reviewing the literature, the following variables were identified as covariates: ambient temperature, maternal age, parity, gravidity, maternal occupation, annual per capita income, passive smoking, history of asthma, preterm birth, gestational diabetes, season of conception, and maternal diet (Multimedia Appendix 2). We used variance inflation factors (VIFs) to detect collinearity among covariates both between individual pollutants and among covariates themselves. A maximum VIF exceeding 10 often suggests that multiple correlations significantly influence least squares estimates. The findings revealed that when all covariates, including temperature, were incorporated, the VIF for PM_2.5_ exceeded 10 in all 3 trimesters, as well as during the saccular and alveolar stages. However, when temperature was excluded from the covariates, all VIFs for PM_2.5_ were below 10 except for the third trimester (Multimedia Appendices 3-5).

### Statistical Analysis

We conducted a comparison of characteristics between the case and control groups using a chi-square test. Pearson correlation was applied to examine correlations between air pollutants and temperature (Multimedia Appendix 4). The mutual effects of air pollution mixtures on asthma/wheezing in children were analyzed using the quantile g-computation (QG-comp) model. QG-comp is used for the impact analysis of exposure mixtures, where it evaluates the exposure as a whole rather than as individual components in its calculations. The QG-comp model combines the simplicity of weighted quantile sum regression inference with the flexibility of the g-calculation method for causal effect estimation. Unlike traditional methods, QG-comp does not necessitate that the direction of the effect is consistent between the exposure variable and the outcome. Moreover, it can capture the effects of all pollutant exposures, 1 quartile increase at a time [26]. The model used the Cox proportional hazards model as the base model and established quartiles of simultaneous increases in all air pollutant concentrations. Hazard ratios (HRs) along with 95% CIs were reported. In addition, the positive or negative weight of each pollutant was documented in each QG-comp model.

In the sensitivity analyses, we narrowed our focus to children with clear information on the feeding method. Furthermore, we adjusted for children’s feeding methods in both prenatal and postnatal QG-comp models for this subgroup to mitigate the potential confounding effect of the feeding method on childhood asthma/wheezing. To assess whether the COVID-19 outbreak in early 2020 and subsequent public health interventions targeting COVID-19 might introduce confounding factors, we conducted sensitivity analyses by limiting the data to the period before the COVID-19 outbreak. In addition, in our sensitivity analyses, we adjusted for air pollutants in the preceding periods in the model for that specific period. This adjustment was made to account for the cumulative effects of early developmental exposure.

All analyses were performed using R 4.2.2 (R Development Core Team 2019 [27]). All statistical tests were conducted using a 2-sided approach.

## Results

### Characteristics of Study Participants

Of the 3725 children included in this study, 392 (10.52%) were diagnosed with asthma/wheezing, with a mean age of onset of 1.48 (SD 0.89) years. At the conclusion of the follow-up period, the mean age of the children was 3.2 (SD 0.8) years, and a total of 14,982 person-years were successfully followed up for all study participants. These included 1984 (53.26%) boys and 1741 (46.74%) girls, with a prevalence of asthma/wheezing of 13.16% (261/1984) in boys compared with 7.52% (131/1741) in girls. The majority of mothers were engaged in commercial activities (2008/3725, 53.91%). Demographic information, the number of asthma cases, and prevalence rates stratified by covariates are presented in Table 1.

**Table 1:** General characteristics of the study children.

Characteristics (variables)		Total (n=3725)	Asthma/wheezing					Chi-square/*t* test (*df*)			*P* value				
			Yes (n=392)		No (n=3333)										
**Sex of children, n (%)**									30.63 (1)			<.001			
	Boys	1984 (53.26)	261 (13.16)		1723 (86.84)										
	Girl	1741 (46.74)	131 (7.52)		1610 (92.48)										
**Maternal age (years), n (%)**									7.19 (3)			.07			
	18-25	193 (5.18)	22 (11.40)		171 (88.60)										
	26-30	1253 (33.64)	124 (9.90)		1129 (90.10)										
	31-35	1304 (35.01)	159 (12.19)		1145 (87.81)										
	>35	975 (26.17)	87 (8.92)		888 (91.08)										
**Maternal occupation, n (%)**									12.94 (6)			.04			
	Manual worker	183 (4.91)	22 (12.02)		161 (87.98)										
	Government official and clerk	78 (2.09)	12 (15.38)		66 (88.62)										
	Housewife	372 (9.99)	43 (11.56)		329 (88.44)										
	Unemployment	278 (7.46)	43 (15.47)		235 (84.53)										
	Technician	689 (18.50)	65 (9.43)		624 (90.57)										
	Business (commercial activities)	2008 (53.91)	193 (9.61)		1815 (90.39)										
	Others	117 (3.14)	14 (11.97)		103 (88.03)										
**Parity, n (%)**									7.86 (2)			.02			
	1	1132 (30.39)	96 (8.48)		1036 (91.52)										
	2	2220 (59.60)	249 (11.22)		1971 (88.78)										
	≥3	373 (10.01)	47 (12.60)		326 (87.40)										
**Gravidity, n (%)**									3.84 (3)			.28			
	1	1104 (29.64)	101 (9.15)		1003 (90.85)										
	2	1432 (38.44)	163 (11.38)		1269 (88.62)										
	3	792 (21.26)	82 (10.35)		710 (89.65)										
	≥4	397 (10.66)	46 (11.59)		351 (88.41)										
**Yearly income per capita (×1000 Yuan^a^), n (%)**									N/A^b^			.43^c^			
	<30	193 (5.18)	22 (11.40)		171 (88.60)										
	30~	2228 (59.81)	248 (11.13)		1980 (88.87)										
	100~	1088 (29.21)	100 (9.19)		988 (90.81)										
	≥200~	178 (4.78)	17 (9.55)		161 (90.45)										
	Refused to answer/missing	38 (1.02)	5 (13.16)		33 (86.84)										
**Feeding method, n (%)**									13.05 (3)			.01			
	Artificial feeding	400 (10.74)	60 (15.00)		340 (85.00)										
	Breastfeeding	449 (12.05)	45 (10.02)		404 (89.98)										
	Mixed feeding	431 (11.57)	32 (7.42)		399 (92.58)										
	Refused to answer/missing	2445 (65.64)	255 (10.43)		2190 (89.57)										
**Passive smoking, n (%)**									0.49 (1)			.49			
	Yes	2599 (69.77)	267 (10.27)		2332 (89.73)										
	No	1126 (30.23)	125 (11.10)		1001 (88.90)										
**Vegetable consumption (time/week), n (%)**									3.73 (3)			.29			
	≤7	345 (9.26)		35 (10.14)		310 (89.86)									
	8-14	1313 (35.25)		122 (9.29)		1191 (90.71)									
	≥15	2005 (53.83)		228 (11.37)		1777 (88.63)									
	Refused to answer/missing	62 (1.66)		7 (11.29)		55 (88.71)									
**Fruit consumption (time/week), n (%)**									4.70 (3)			.20			
	≤5	271 (7.28)		24 (8.86)		247 (91.14)									
	6-7	3041 (81.64)		334 (10.98)		2707 (89.02)									
	≥8	343 (9.21)		26 (7.58)		317 (92.42)									
	Refused to answer/missing	70 (1.88)		8 (11.43)		62 (88.57)									
**History of gestational diabetes mellitus, n (%)**									N/A			.59^c^			
	No	3516 (94.39)		368 (10.47)		3148 (89.53)									
	Yes	197 (5.29)		22 (11.17)		175 (88.83)									
	Refused to answer/missing	12 (0.32)		2 (16.67)		10 (83.33)									
**History of maternal asthma, n (%)**									0.17 (1)			.68			
	No	3546 (95.19)		371 (10.46)		3175 (89.54)									
	Yes	179 (4.81)		21 (11.73)		158 (88.27)									
**Conception season, n (%)**									3.03 (1)			.08			
	Warm (May to October)	1775 (47.65)		170 (9.58)		1605 (90.42)									
	Cold (November to April)	1950 (52.35)		222 (11.38)		1728 (88.62)									
**Premature, n (%)**									1.93 (1)			.17			
	No	3520 (94.50)		364 (10.34)		3156 (89.66)									
	Yes	205 (5.50)		28 (13.66)		177 (86.34)									
Age of children at end of follow-up (years), mean (SD)		3.20 (0.78)		1.48 (0.89)		3.41 (0.44)		42.21^d^ (3723)			<.001				
Gestation age of children at birth (ie, gestational weeks), mean (SD)		39.74 (2.08)		39.65 (1.61)		39.75 (2.13)		1.12^d^ (3273)			.26				

^a^1 yuan=US $0.14.

^b^N/A: not applicable.

^c^Fisher exact test.

^d^*t*-test for 2 independent samples (paired, 2-tailed).

### Associations Between Maternal Ambient Pollutant Exposure and Childhood Wheezing Risk

**Figure 2:**
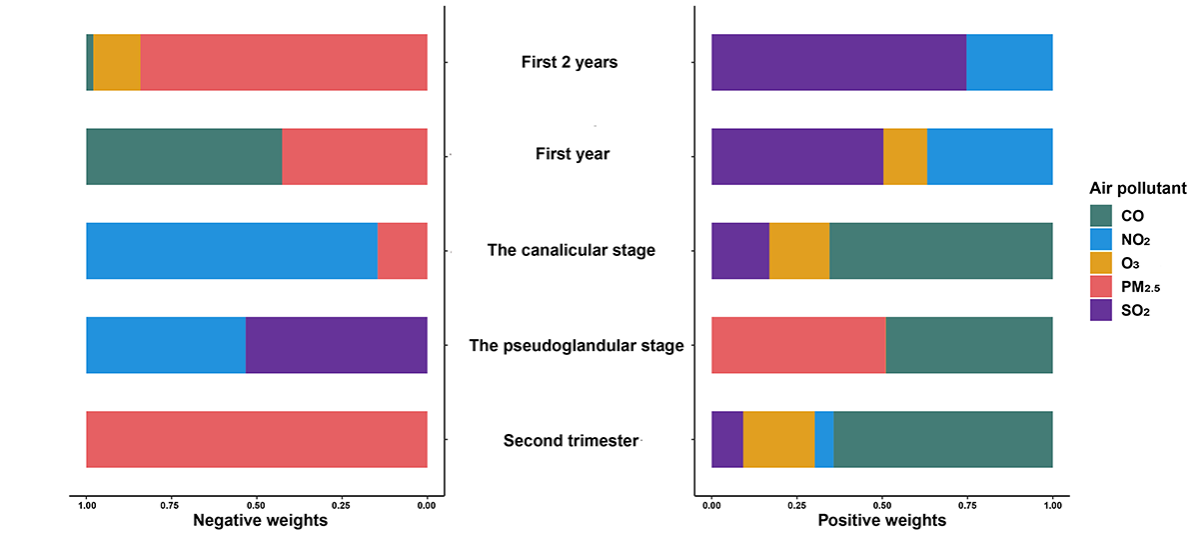
Weights of the proportion of the positive or negative partial effect of each air pollutant. CO: carbon monoxide; NO_2_: nitrogen dioxide; O_3_: ozone; PM_2.5_: particulate matter 2.5; SO_2_: sulfur dioxide.

Table 2 displays the risk of asthma/wheezing in children exposed to mixed air pollutants during pregnancy. In the adjustment model, each quartile increase in exposure to mixed air pollutants during the second trimester was linked to an adjusted HR of 1.24 (95% CI 1.04-1.47), with CO contributing the most positively (64.28%) to the joint effect (Figure 2). Following staging by lung development, each additional quartile of exposure to mixed air pollutants during the pseudoglandular and canalicular stages was associated with HRs of 1.24 (95% CI 1.03-1.51) and 1.23 (95% CI 1.01-1.51), respectively. During the pseudoglandular stage, PM_2.5_ (50.98%) and CO (48.92%) made the greatest positive contributions, while CO made the largest positive contribution during the canalicular stage (65.50%; Figure 2).

**Table 2:** Associations (HR^a^ and 95% CI) of per quartile increase in a mixture of air pollutants during the pregnancy period with the risk of childhood asthma/wheezing.

Association		Model 1^b^, crude HR (95% CI)	Model 2^c^, adjusted HR (95% CI)
Whole pregnancy		1.05 (0.92-1.21)	1.11 (0.94-1.30)
**Grouped by clinical staging**			
	First trimester	1.19 (0.99-1.42)	1.22 (1.00-1.48)^d^
	Second trimester	1.16 (0.99-1.36)	1.24 (1.04-1.47)^d^
	Third trimester	1.10 (0.92-1.28)	1.10 (0.89-1.36)^d^
**Grouped by lung development**			
	The embryonic stage	1.03 (0.87-1.22)	1.02 (0.85-1.24)
	The pseudoglandular stage	1.15 (0.97-1.37)	1.24 (1.03-1.51)
	The canalicular stage	1.14 (0.97-1.34)	1.23 (1.01-1.50)
	The saccular stage	0.99 (0.84-1.16)	1.08 (0.89-1.32)^d^
	The alveolar stage	1.04 (0.88-1.22)	0.98 (0.82-1.18)^d^

^a^HR: hazard ratio.

^b^Crude model.

^c^Adjusted for temperature, maternal age, gravidity, parity, maternal occupation, yearly income per capita, passive smoking, maternal diet, history of asthma, gestational diabetes mellitus, preterm birth, and season of conception.

^d^There is a potential collinearity between temperature and air pollutants. The adjusted covariates do not include temperature.

### Associations Between Child Ambient Pollutants Exposure and Childhood Wheezing Risk

**Figure 3:**
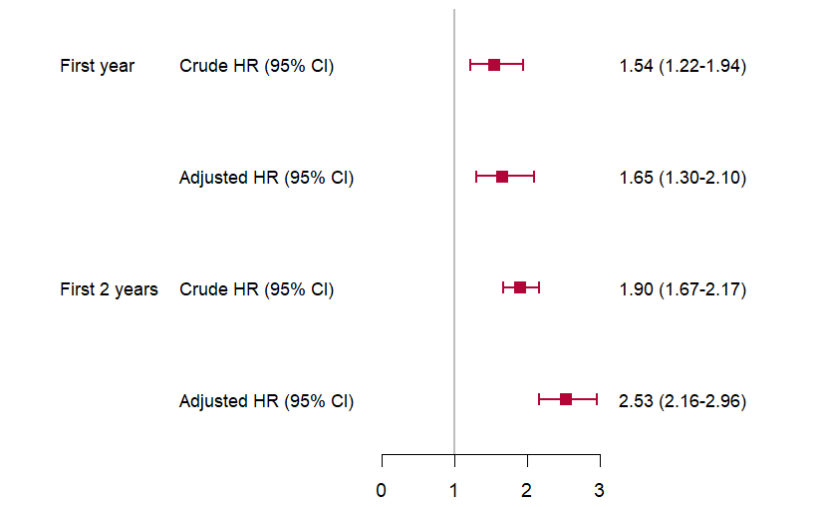
Associations (HR, 95%CI) of per quartile increase in the mixture of air pollutants during the childhood with the risk of childhood asthma/wheezing. HR: hazard ratio.

The adjusted HRs for childhood asthma and wheezing were 1.24 (95% CI 1.30-2.10) and 1.23 (95% CI 2.16-2.97), respectively, for each quartile increase in air pollutants during the first year and first 2 years of childhood. Notably, SO_2_ made the largest positive contribution in both periods, accounting for 50.30% and 74.70%, respectively. Another significant contributor is NO_2_, accounting for 36.8% and 25.3% in the first year and first 2 years of childhood, respectively (Figures 2 and 3).

### Sensitivity Analyses

We conducted sensitivity analyses in a subgroup of children with clear information on the feeding method and restricted the data to the period before the COVID-19 outbreak. Adjusting for the feeding method and the COVID-19 outbreak did not substantially alter the associations of prenatal and postnatal exposure to a mixture of air pollutants with the risk of childhood wheezing (Table 3). Similarly, the relationship between air pollutants and childhood asthma/wheezing did not change significantly after adjusting for air pollutants in the preceding periods in the model for that specific period (Table 4).

**Table 3:** Sensitivity analyses for subgroups of children with clear information on the feeding method and adjusted for cut-off date.

Analysis	Whole pregnancy, HR^a^ (95% CI)	The first 2 years, HR (95% CI)
Model 1^b^	1.32 (1.00-1.77)	2.39 (1.83-3.12)
Model 2^c^	1.34 (1.01-1.78)	2.42 (1.85-3.15)
Model 3^d^	1.11 (0.94-1.30)	2.53 (2.16-2.96)
Model 4^e^	1.16 (0.98-1.37)	2.68 (2.27-3.18)

^a^HR: hazard ratio.

^b^Adjusted for the feeding mode, body temperature, maternal age, maternity, maternal occupation, per capita annual income, passive smoking, maternal diet, history of asthma, gestational diabetes mellitus, preterm birth, and season of conception among those with clear information on the feeding method.

^c^Adjusted for confounders in model 1 except for the feeding method.

^d^The cohort’s follow-up cut-off date was set at July 31, 2020.

^e^The cohort’s follow-up cut-off date was set at December 31, 2019.

**Table 4:** Association between air pollutants and childhood asthma/wheezing after adjusting for preceding cyclic pollutants.

Association			Model 1, HR^a^ (95% CI)	Model 2^b^, adjusted HR (95% CI)	
**Grouped by clinical staging**					
		First trimester	1.22 (1.00-1.48)^c^	N/A^d^	
		Second trimester	1.24 (1.04-1.47)^c^	1.41 (1.13-1.77)	
		Third trimester	1.10 (0.89-1.36)^c^	1.04 (0.83-1.31)	
**Grouped by lung development**					
		The embryonic stage	1.02 (0.85-1.24)^e^	N/A	
		The pseudoglandular stage	1.24 (1.03-1.51)^e^	1.45 (1.13-1.86)	
		The canalicular stage	1.23 (1.01-1.50)^e^	1.30 (1.03-1.64)	
		The saccular stage	1.08 (0.89-1.32)^c^	1.03 (0.83-1.28)	
		The alveolar stage	0.98 (0.82-1.18)^c^	0.94 (0.77-1.15)	
**Postnatal**					
	First year		1.65 (1.30-2.10)^e^	1.77 (1.36-2.29)	
	First 2 years		2.53 (2.16-2.96)^e^	3.66 (3.05-4.38)	

^a^HR: hazard ratio.

^b^We adjusted the pollutants for the period preceding each period based on model 1. For example, air pollutants from the first trimester were included as covariates in the model for the second trimester, and air pollutants from both the first and second trimesters were included as covariates in the model for the third trimester. Air pollutants from the embryonic stage were added to the covariates in the model for the pseudoglandular stage, while contaminants from both the embryonic and pseudoglandular stages were added to the covariates in the model for the canalicular stage to adjust the model. Contaminants from the previous 3 periods were added to the covariates in the saccular stage model to adjust the model, and contaminants from the previous 4 periods were added to the covariates in the model for the alveolar stage to adjust the model. We also added pollutant exposure throughout pregnancy as a covariate in the first year and first 2 postnatal year models for adjustment.

^c^There is a potential collinearity between temperature and air pollutants. The adjusted covariates do not include temperature.

^d^N/A: not applicable.

^e^Adjusted for temperature, maternal age, gravidity, parity, maternal occupation, yearly income per capita, passive smoking, maternal diet, history of asthma, gestational diabetes mellitus, premature, and season of conception.

## Discussion

### Principal Findings

In this prospective cohort study, we assessed the effects of exposure to 5 mixed air pollutants, including PM_2.5_, SO_2_, NO_2_, CO, and O_3_, during the first 1000 days of life on the incidence of asthma/wheezing in children. We observed positive associations between exposure to mixed air pollutants and the incidence of asthma/wheezing. Furthermore, exposure to mixed air pollutants during the second trimester, particularly during the pseudoglandular and canalicular stages, as well as in the first 2 years of life, may have a greater impact on the risk of childhood asthma/wheezing. These findings suggest potential critical exposure windows for air pollution. They extend our understanding of the adverse effects of air pollution exposure during early life on children’s respiratory health and provide suggestive information for implementing protective measures to mitigate the adverse effects of air pollution on children’s health.

Several epidemiological studies have also reported associations between maternal exposure to single or multiple air pollutants during pregnancy and the risk of childhood asthma, which aligns with our findings. For instance, a Canadian birth cohort study investigated the impact of perinatal air pollution exposure on asthma exacerbation in children, revealing that early-life exposure to higher concentrations of CO, NO_x_, PM_10_, and SO_2_ elevates the risk of asthma in preschool children [28]. However, the aforementioned study took place earlier than this study and involved a city with lower temperatures and air pollutant concentrations compared with Guangzhou. Another cohort study from China demonstrated that each interquartile range increment of maternal exposure to NO_2_ during pregnancy was positively associated with childhood asthma [29]. The study was conducted in 3 northern Chinese cities (Urumqi, Beijing, and Taiyuan) and 4 southern cities (Nanjing, Shanghai, Chongqing, and Changsha). However, the 3 northern cities typically experience lower temperatures compared with Guangzhou. Nevertheless, both studies reached similar conclusions to our study, despite significant differences in terms of the number of years studied, air temperature, and air pollutant concentrations between the 2 studies and this study.

Subgroup analyses indicated that the second trimester, specifically the pseudoglandular stage and the canalicular stage, may represent critical exposure windows for mixed air pollution–induced asthma in children. This finding is consistent with previous studies [21,30,31]. The Asthma Coalition for Community, Environmental, and Social Stress (ACCESS) project in the United States discovered that maternal exposure to higher PM_2.5_ concentrations may elevate the risk of asthma attacks in offspring at 6 years of age, with the gestational period between 16 and 25 weeks potentially holding particular significance [21]. Another cohort study from Canada similarly demonstrated a significant association between maternal midgestational exposure to PM_2.5_ and NO_2_ and the incidence of asthma in children [30].

During the pseudoglandular stage, which occurs during GWs 6-16, all potentially conducting airways are formed, and the emergence of alveolar contours occurs. This stage is characterized by the significant development of fine bronchial structures. By GW 17, the major components of the lung are mostly formed, with the exception of the gas exchange component. During the canalicular stage, which spans GWs 16-24, early development of the lung parenchyma occurs. This stage is characterized by the expansion of the lumen of the bronchi and terminal fine bronchioles, as well as the initiation of thin-walled terminal vesicle formation at the ends of the respiratory fine bronchioles [22,32]. By the end of the canalicular stage, all conducting airways have emerged, allowing respiration and gas exchange to commence [33]. This indicates that during the pseudoglandular and canalicular stages, the developing lungs may be more susceptible to inhaled air pollutants, potentially leading to the development of asthma in childhood.

We also observed that exposure to mixed air pollutants during the first 2 years of life significantly elevated the risk of asthma/wheezing, suggesting that this period may represent another important window. This finding aligns with previous studies, such as the one [10] that investigated the association between air pollution and asthma development in all children born between 1999 and 2000 in southwestern British Columbia, Canada. The study assessed air pollution exposure in quartiles and discovered that children exhibited an increased odds ratio for asthma when exposed to higher quartiles of CO, NO, NO_2_, PM_10_, and SO_2_ in the first year of life [10]. Furthermore, a prospective cohort study from Copenhagen identified air pollution as being associated with wheezing symptoms in children. They found a significant effect of NO_2_ on asthma symptoms in infants 3 years after birth and also noted a significant correlation between PM_10_, CO concentrations, and wheezing symptoms in infants (0-1-year olds) [34]. A study conducted in Changsha, China, similarly demonstrated that postnatal exposure to outdoor industrial and traffic air pollutants, including PM_10_, SO_2_, and NO_2_, was significantly associated with an increased risk of asthma in children [35].

Studies have indicated that infants are particularly vulnerable to the effects of air pollutants due to their incomplete development of respiratory and immune systems [36]. Furthermore, alveolarization primarily occurs after birth, with approximately 85% of alveoli developing postnatally. Lung volume doubles by 6 months of age and triples by 1 year of age [37]. Collectively, these findings suggest that children’s respiratory systems continue to rapidly develop after birth, potentially rendering their lungs more sensitive to inhaled air pollutants.

In addition, our weighted analyses revealed that air pollutants at various stages may exert distinct impacts on the development of asthma in children. During the second trimester and the canalicular period, CO emerged as the largest contributor to the development of asthma/wheezing in children. Equally significant contributors during the pseudoglandular stage were CO and PM_2.5_. In childhood, the air pollutants contributing the most were SO_2_ and NO_2_.

Many previous studies investigating the relationship between air pollutants and respiratory diseases have placed particular emphasis on PM_2.5_ [38,39]. These studies suggest that the pathogenic mechanism of PM_2.5_ is primarily associated with oxidative stress. This stress can either cross the placental barrier and directly affect the fetus or induce systemic inflammation in the mother, leading to a reduction in the supply of nutrients and oxygen to the fetus, thereby affecting fetal lung function [16]. However, our study proposes that CO may be a more significant contributor to air pollutants affecting lung development in pregnant children than PM_2.5_.

CO is a highly soluble, nonirritant gas that easily enters the bloodstream through the alveoli, capillaries, and placental barrier. Once in the bloodstream, it competes with oxygen and binds to hemoglobin, resulting in tissue hypoxia [40]. Hypoxia in maternal tissues can decrease the amount of gases carried by the fetus, potentially affecting the development of fetal lung function and possibly leading to the development of childhood asthma. However, there has been relatively less focus on its association with the development of asthma in children in developing countries, and the results of the few studies conducted have been inconsistent [41]. Therefore, it is recommended that future research on the relationship between CO and childhood asthma should be intensified to further clarify the association and the pathogenesis of the disease.

SO_2_ is primarily released into the atmosphere through the combustion of fossil fuels and is highly soluble in water. Its effects are primarily harmful to the upper respiratory tract. SO_2_ can cause severe airway constriction, which may be related to its pathogenic mechanism [41]. Andersson et al [42] reported an HR of 5.8 (95% CI 2.6-13) for asthma among individuals reporting inhalation of SO_2_ gas compared with unexposed individuals following a survey of sulfite mill workers. In addition, several studies have demonstrated a connection between SO_2_ and the development of asthma in children [43,44]. NO_2_ is among the main components of TRAP. Similarly, the association between NO_2_ and asthma has been affirmed by several studies [33,45,46]. Health Canada has also proposed a causal relationship between exposure to current levels of ambient NO_2_ and increased asthma-related morbidity [47]. NO_2_ is insoluble in water; it dissolves in the most distal airways and alveoli after inhalation. Subsequently, reactive oxygen and nitrogen species are produced, which can induce oxidative stress and damage to the respiratory tract. This mechanism potentially contributes to the development of asthma [41].

### Limitations

There are several limitations that need to be acknowledged. First, due to the challenges associated with early diagnosis of asthma, there may be cases that do not present to the hospital after the onset of the disease, leading to missed diagnoses. In addition, some of the wheezing cases observed may not have been asthma, potentially resulting in misclassification of outcome measures. By contrast, the small number of diagnosed cases of asthma led us to combine asthma and wheezing into a single outcome. This approach prevented us from accurately estimating the effects of outdoor air pollution exposure on asthma incidence. Third, our study participants were sourced from only 1 hospital, and the administrative area covered by the hospital’s services (Panyu District, Guangzhou) differs somewhat from the demographic composition of the entire city of Guangzhou and of China (Multimedia Appendix 1). This aspect also imposes limitations on the generalizability of our results. Finally, we conducted a passive survey of children during their visits to a pediatric clinic, pediatric outpatient department, or emergency department. Children who did not attend the hospital were lost to follow-up, potentially leading to an underestimation of the effect of air pollutants.

### Conclusion

This prospective cohort study offers new evidence suggesting a potential association between exposure to ambient mixed air pollutants during the first 1000 days of life and childhood asthma/wheezing. The second trimester, particularly the pseudoglandular stage and the canalicular stage, as well as the first 2 years after birth, may represent critical susceptibility periods for this exposure window. Our study is a cohort study, and therefore, firm causal inferences cannot be made. However, by incorporating findings from previous studies, our research contributes to a broader understanding of the adverse respiratory effects of outdoor air pollution exposure. This knowledge can empower clinicians and pregnant women to take proactive measures to reduce air pollution exposure, particularly during vulnerable windows of susceptibility. Such preventive measures are crucial for the prevention of childhood asthma.

## Appendix

Multimedia Appendix 1 General demographic information.

Multimedia Appendix 2 Definition of covariates.

Multimedia Appendix 3 VIF was used to check the collinearity of each component in the model. VIF: variance inflation factor.

Multimedia Appendix 4 Correlation between pollutants and temperature.

Multimedia Appendix 5 VIF for each pollutant in the prenatal and postnatal reciprocal adjustment models. VIF: variance inflation factor.

## Abbreviations

ACCESS: Asthma Coalition for Community, Environmental, and Social Stress
CO: carbon monoxide
GW: gestational week
HR: hazard ratio
ICD-10: 10th edition of the International Classification of Diseases
NO_2_: nitrogen dioxide
O_3_: ozone
PEOH: Prenatal Environments and Offspring Health
PM_2.5_: particulate matter 2.5
QG-comp: quantile g-computation
SO_2_: sulfur dioxide
ST-LUR: spatiotemporal land use regression
TRAP: traffic-related air mixture pollutant
VIF: variance inflation factor

## Data Sharing Statement

The data that support the findings of this study will be available upon reasonable request to the corresponding author of the study.
