# Supplementary material for: Mutual Associations of Exposure to Ambient Air Pollutants in the First 1000 Days of Life With Asthma/Wheezing in Children: Prospective Cohort Study in Guangzhou, China
Source: JMIR Public Health Surveill. 2024 Apr 17;10:e52456. doi: 10.2196/52456 (PMC11063886; doi:10.2196/52456)
Supplement: Multimedia Appendix 1 [file publichealth_v10i1e52456_app1.docx]

This supplementary material has been provided by the authors to give readers additional information about their works.

| District | 0-14y | | | 15-64y | | | 65y~ | | | Total | | |
| --- | --- | --- | --- | --- | --- | --- | --- | --- | --- | --- | --- | --- |
|  | Male | Female | Sex ratio | Male | Female | Sex ratio | Male | Female | Sex ratio | Male | Female | Sex ratio |
| China | 134593579 | 118790359 | 113.3 | 496312454 | 469447052 | 105.7 | 90510361 | 100124919 | 90.4 | 721416394 | 688362330 | 104.8 |
| Guangzhou | 1397533 | 1192656 | 117.2 | 7794435 | 6831648 | 114.1 | 674037 | 786296 | 85.7 | 9866005 | 8810600 | 111.98 |
| Panyu | 190003 | 164422 | 115.6 | 1145694 | 1004954 | 114.0 | 69182 | 84142 | 82.2 | 1404879 | 1253518 | 112.07 |

Note: ^a^ Data from the Seventh Population Census
