# Supplementary material for: Mutual Associations of Exposure to Ambient Air Pollutants in the First 1000 Days of Life With Asthma/Wheezing in Children: Prospective Cohort Study in Guangzhou, China
Source: JMIR Public Health Surveill. 2024 Apr 17;10:e52456. doi: 10.2196/52456 (PMC11063886; doi:10.2196/52456)
Supplement: Multimedia Appendix 2 [file publichealth_v10i1e52456_app2.docx]

| Name | Definition |
| --- | --- |
| Week of gestation | The week of gestation is estimated from the first day of the last menstrual period, based on the date of the last menstrual period reported by the pregnant woman or the ultrasound, in case of discrepancy between the two, the ultrasound result will prevail. |
| Artificial feeding | It is the replacement of breast milk with other formulae if the baby cannot be breastfed. |
| Mixed feeding | Artificial feeding and breastfeeding at the same time. |
| Gestational diabetes | According to the IADPSG criteria, a pregnant woman is diagnosed with gestational diabetes if she meets one or more of the following criteria: (a) fasting glucose ≥ 5.1 mmol l^-1^ or (b) 75g oral glucose tolerance test (OGTT) with baseline plasma glucose ≥ 5.1 mmol l^-1^, 1-hour plasma glucose ≥ 10.0 mmol l^-1^, or 2-hour plasma glucose ≥ 8.5 mmol l^-1^ |
| Premature | Delivery at 28 weeks of pregnancy but less than 37 weeks |
| Parity | Number of times a woman who is more than 24 weeks pregnant has given birth to a foetus, regardless of whether the child was born alive or stillborn. |
| Gravidity | The number of pregnancies a woman has had, including the number of deliveries, miscarriages and induced abortions. |
| Passive smoking | Pregnant women who are not smokers themselves but are exposed to second-hand smoke for at least one minute a day during pregnancy; |
| TM(Temperature ) | Temperature data from Guangdong Meteorological Service, Weekly mean corresponding to air pollutant values are used. |
