# Supplementary material for: Mutual Associations of Exposure to Ambient Air Pollutants in the First 1000 Days of Life With Asthma/Wheezing in Children: Prospective Cohort Study in Guangzhou, China
Source: JMIR Public Health Surveill. 2024 Apr 17;10:e52456. doi: 10.2196/52456 (PMC11063886; doi:10.2196/52456)
Supplement: Multimedia Appendix 3 [file publichealth_v10i1e52456_app3.docx]

**Multimedia Appendix 3.** Variance inflation factor (VIF) was used to check the collinearity of each component in the model.

|  | Model1^a^ | | | | |  | Model2^b^ | | | | |  | Model3^c^ | | | | |
| --- | --- | --- | --- | --- | --- | --- | --- | --- | --- | --- | --- | --- | --- | --- | --- | --- | --- |
|  | PM_2.5_ | SO_2_ | NO_2_ | CO | O_3_ |  | PM_2.5_ | SO_2_ | NO_2_ | CO | O_3_ |  | PM_2.5_ | SO_2_ | NO_2_ | CO | O_3_ |
| Whole pregnancy | 2.45 | 1.36 | 2.17 | 1.41 | 1.06 |  | 2.67 | 1.46 | 2.72 | 1.46 | 1.72 |  | 4.39 | 1.46 | 2.71 | 1.46 | 2.70 |
| **Grouped by clinical staging** | | | | | |  |  |  |  |  |  |  |  |  |  |  |  |
| First trimester | 6.85 | 2.38 | 5.32 | 3.05 | 1.81 |  | 9.17 | 2.48 | 6.89 | 3.15 | 1.95 |  | 10.01 | 2.83 | 7.18 | 3.15 | 4.50 |
| Second trimester | 6.91 | 1.70 | 4.96 | 3.26 | 1.34 |  | 7.12 | 1.94 | 6.23 | 3.20 | 1.72 |  | 10.29 | 2.01 | 6.49 | 3.32 | 3.01 |
| Third trimester | 8.19 | 1.42 | 6.69 | 3.34 | 1.55 |  | 10.71 | 1.58 | 8.34 | 3.49 | 1.64 |  | 18.26 | 1.99 | 9.19 | 3.59 | 2.69 |
| **Grouped by respiratory development** | | | | | |  |  |  |  |  |  |  |  |  |  |  |  |
| The embryonic stage | 6.19 | 2.09 | 6.05 | 2.92 | 1.89 |  | 7.51 | 2.35 | 7.88 | 3.24 | 1.95 |  | 8.66 | 3.10 | 7.59 | 3.17 | 3.51 |
| The pseudoglandular stage | 6.23 | 2.28 | 5.01 | 2.97 | 1.67 |  | 6.48 | 2.32 | 5.76 | 2.97 | 1.92 |  | 8.27 | 2.77 | 6.20 | 3.04 | 3.15 |
| The canalicular stage | 6.72 | 1.69 | 5.17 | 3.41 | 1.33 |  | 6.66 | 1.74 | 5.39 | 3.32 | 1.66 |  | 8.42 | 1.96 | 5.75 | 3.39 | 2.28 |
| The saccular stage | 7.41 | 1.53 | 5.97 | 3.48 | 1.45 |  | 9.45 | 1.77 | 7.72 | 3.55 | 1.57 |  | 15.63 | 2.02 | 8.08 | 3.60 | 2.47 |
| The alveolar stage | 8.13 | 1.69 | 6.65 | 3.37 | 1.72 |  | 10.81 | 1.72 | 7.15 | 3.50 | 2.31 |  | 14.91 | 2.05 | 7.14 | 3.62 | 2.90 |
| **Postnatal** |  |  |  |  |  |  |  |  |  |  |  |  |  |  |  |  |  |
| First-year | 1.85 | 1.97 | 1.99 | 1.44 | 1.92 |  | 1.99 | 2.00 | 2.33 | 1.88 | 2.03 |  | 2.09 | 2.00 | 2.74 | 1.90 | 2.38 |
| First two years | 5.38 | 3.57 | 2.74 | 2.19 | 1.20 |  | 5.28 | 3.66 | 3.01 | 2.51 | 1.16 |  | 5.58 | 3.51 | 4.12 | 2.71 | 1.43 |

Note: ^a^ Crude model

^b^ Adjustment for maternal age, gravidity, parity, maternal occupation, yearly income per capita, passive smoking, maternal diet, history of asthma, and gestational diabetes mellitus, premature, and season of conception.

^c^ Adjustment for temperature, maternal age, gravidity, parity, maternal occupation, yearly income per capita, passive smoking, feeding method, maternal diet, history of asthma, and gestational diabetes mellitus, premature, and season of conception.

**Multimedia Appendix 3.1.** Variance inflation factor (VIF) was used to check the collinearity of each component in the model2.(continued )

| ^d^ | ① | ② | ③ | ④ | ⑤ | ⑥ | ⑦ | ⑧ | ⑨ | ⑩ | ⑪ | ⑫ |
| --- | --- | --- | --- | --- | --- | --- | --- | --- | --- | --- | --- | --- |
| Whole pregnancy | 1.34 | 1.05 | 2.09 | 1.65 | 1.05 | 1.11 | 1.05 | 1.05 | 1.12 | 1.16 | 1.41 | 1.02 |
| **Grouped by clinical staging** |  | | | | | | | | | | | |
| First trimester | 1.34 | 1.05 | 1.85 | 1.54 | 1.05 | 1.08 | 1.05 | 1.05 | 1.09 | 1.12 | 2.17 | 1.02 |
| Second trimester | 1.34 | 1.05 | 2.03 | 1.60 | 1.06 | 1.11 | 1.05 | 1.05 | 1.11 | 1.15 | 1.59 | 1.03 |
| Third trimester | 1.34 | 1.05 | 1.94 | 1.58 | 1.05 | 1.07 | 1.05 | 1.05 | 1.10 | 1.12 | 2.52 | 1.02 |
| **Grouped by respiratory development** | | | | | | | | | | | | |
| The embryonic stage | 1.34 | 1.05 | 1.77 | 1.51 | 1.04 | 1.09 | 1.05 | 1.05 | 1.09 | 1.12 | 1.73 | 1.02 |
| The pseudoglandular stage | 1.34 | 1.04 | 1.85 | 1.54 | 1.05 | 1.09 | 1.05 | 1.05 | 1.09 | 1.12 | 1.18 | 1.17 |
| The canalicular stage | 1.33 | 1.05 | 1.92 | 1.58 | 1.05 | 1.11 | 1.05 | 1.05 | 1.10 | 1.14 | 1.20 | 1.19 |
| The saccular stage | 1.34 | 1.05 | 1.97 | 1.59 | 1.05 | 1.10 | 1.05 | 1.05 | 1.09 | 1.13 | 2.01 | 1.02 |
| The alveolar stage | 1.34 | 1.05 | 1.80 | 1.52 | 1.05 | 1.08 | 1.05 | 1.05 | 1.09 | 1.11 | 3.56 | 1.02 |
| **Postnatal** | | | | | | | | | | | | |
| First-year | 1.34 | 1.05 | 1.96 | 1.56 | 1.05 | 1.07 | 1.05 | 1.05 | 1.09 | 1.13 | 1.21 | 1.02 |
| First two years | 1.35 | 1.06 | 1.88 | 1.50 | 1.04 | 1.07 | 1.06 | 1.06 | 1.08 | 1.08 | 1.27 | 1.02 |

Note: ^d^ Covariates

①maternal age、②maternal occupation、③parity、④gravidity、⑤yearly income per capita、⑥vegetable.consumption⑦fruit.consumption

⑧passive smoking、⑨gestational diabetes mellitus、⑩history of asthma、⑪season of conception、⑫premature

**Multimedia Appendix 3.2.** Variance inflation factor (VIF) was used to check the collinearity of each component in the model3.(continued )

|  | TM | ① | ② | ③ | ④ | ⑤ | ⑥ | ⑦ | ⑧ | ⑨ | ⑩ | ⑪ | ⑫ |
| --- | --- | --- | --- | --- | --- | --- | --- | --- | --- | --- | --- | --- | --- |
| Whole pregnancy | 5.33 | 1.34 | 1.05 | 2.11 | 1.65 | 1.05 | 1.12 | 1.05 | 1.05 | 1.12 | 1.16 | 2.05 | 1.02 |
| **Grouped by clinical staging** | | | | | | | | | | | | | |
| First trimester | 9.07 | 1.34 | 1.05 | 1.84 | 1.54 | 1.05 | 1.11 | 1.06 | 1.05 | 1.09 | 1.12 | 2.47 | 1.02 |
| Second trimester | 10.23 | 1.34 | 1.05 | 2.04 | 1.61 | 1.06 | 1.13 | 1.05 | 1.05 | 1.11 | 1.15 | 1.93 | 1.03 |
| Third trimester | 13.05 | 1.34 | 1.05 | 1.94 | 1.58 | 1.05 | 1.08 | 1.05 | 1.05 | 1.09 | 1.12 | 2.53 | 1.02 |
| **Grouped by respiratory development** | | | | | | | | | | | | | |
| The embryonic stage | 4.90 | 1.34 | 1.05 | 1.77 | 1.51 | 1.05 | 1.11 | 1.06 | 1.05 | 1.09 | 1.12 | 1.87 | 1.02 |
| The pseudoglandular stage | 5.90 | 1.36 | 1.05 | 1.89 | 1.55 | 1.05 | 1.12 | 1.06 | 1.05 | 1.09 | 1.12 | 1.18 | 1.18 |
| The canalicular stage | 5.77 | 1.34 | 1.04 | 1.94 | 1.59 | 1.06 | 1.13 | 1.06 | 1.05 | 1.11 | 1.14 | 1.20 | 1.19 |
| The saccular stage | 10.12 | 1.34 | 1.05 | 1.98 | 1.59 | 1.05 | 1.11 | 1.05 | 1.05 | 1.09 | 1.13 | 2.02 | 1.03 |
| The alveolar stage | 6.36 | 1.34 | 1.05 | 1.80 | 1.53 | 1.04 | 1.09 | 1.05 | 1.05 | 1.09 | 1.11 | 3.98 | 1.02 |
| **Postnatal** | | | | | | | | | | | | | |
| First-year | 1.64 | 1.35 | 1.05 | 1.96 | 1.56 | 1.05 | 1.08 | 1.05 | 1.06 | 1.08 | 1.13 | 1.35 | 1.02 |
| First two years | 1.93 | 1.37 | 1.07 | 1.91 | 1.52 | 1.03 | 1.07 | 1.07 | 1.06 | 1.08 | 1.08 | 1.30 | 1.02 |

Note: ^d^ Covariates

①maternal age、②maternal occupation、③parity、④gravidity、⑤yearly income per capita、⑥vegetable.consumption⑦fruit.consumption

⑧passive smoking、⑨gestational diabetes mellitus、⑩history of asthma、⑪season of conception、⑫premature
