# Supplementary figures and images for: Mutual Associations of Exposure to Ambient Air Pollutants in the First 1000 Days of Life With Asthma/Wheezing in Children: Prospective Cohort Study in Guangzhou, China
Source: JMIR Public Health Surveill. 2024 Apr 17;10:e52456. doi: 10.2196/52456 (PMC11063886; doi:10.2196/52456)

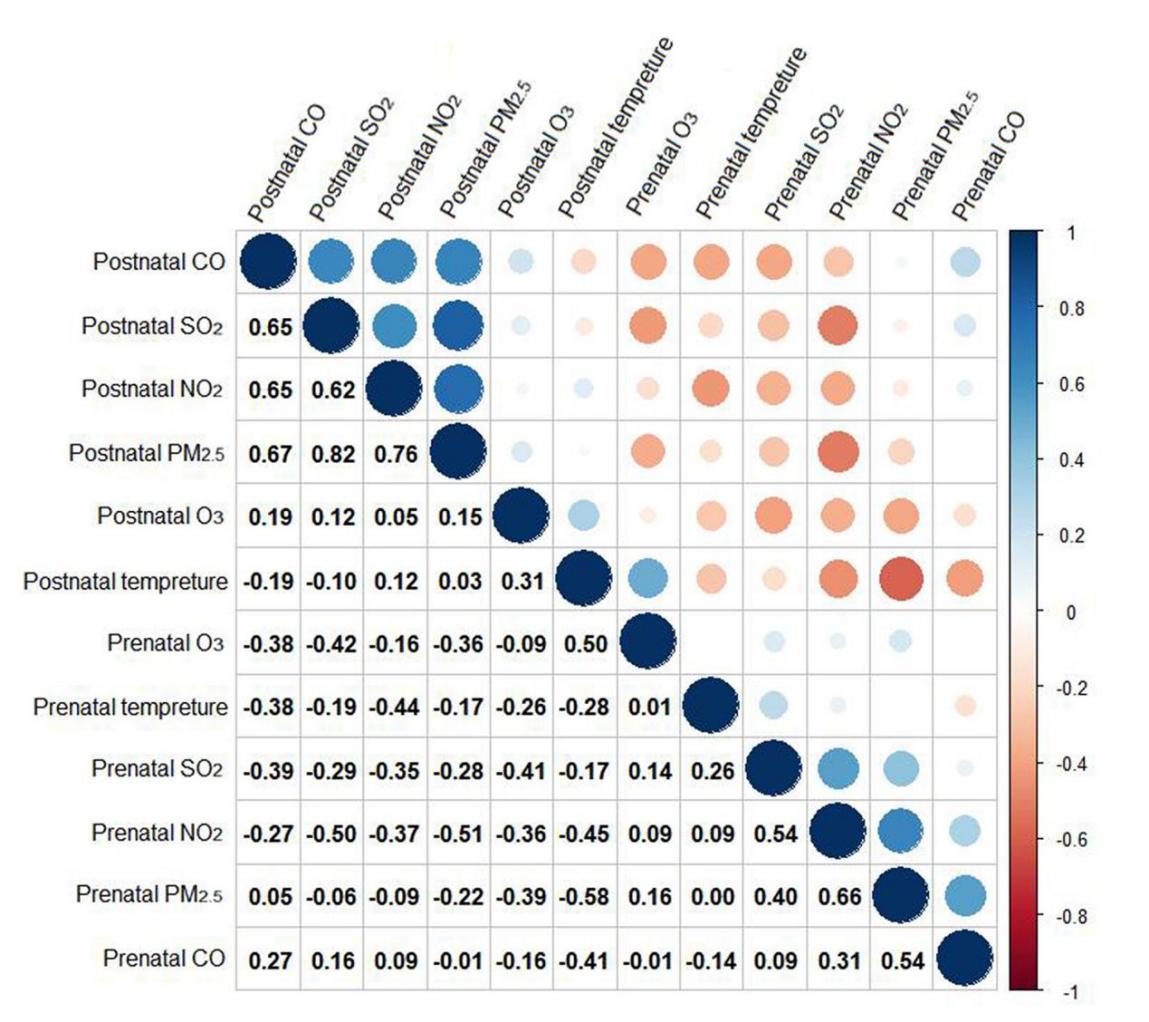

Supplement: Multimedia Appendix 4 [file publichealth_v10i1e52456_app4.docx]
