# Supplementary material for: Mutual Associations of Exposure to Ambient Air Pollutants in the First 1000 Days of Life With Asthma/Wheezing in Children: Prospective Cohort Study in Guangzhou, China
Source: JMIR Public Health Surveill. 2024 Apr 17;10:e52456. doi: 10.2196/52456 (PMC11063886; doi:10.2196/52456)
Supplement: Multimedia Appendix 5 [file publichealth_v10i1e52456_app5.docx]

|  | Model1^a^ | | | | |  | Model2^b^ | | | | |
| --- | --- | --- | --- | --- | --- | --- | --- | --- | --- | --- | --- |
|  | PM_2.5_ | SO_2_ | NO_2_ | CO | O_3_ |  | PM_2.5_ | SO_2_ | NO_2_ | CO | O_3_ |
| Pregnancy | 4.39 | 1.46 | 2.71 | 1.46 | 2.70 |  | 4.93 | 2.12 | 4.18 | 1.89 | 4.60 |
| Postnatal | 5.58 | 3.51 | 4.12 | 2.71 | 1.43 |  | 6.40 | 3.79 | 5.40 | 3.37 | 1.48 |

Note: ^a^ Adjustment for temperature, maternal age, gravidity, parity, maternal occupation, yearly income per capita, passive smoking, feeding method, maternal diet, history of asthma, and gestational diabetes mellitus, premature, and season of conception.

^b^ Mutual adjustment of prenatal and postnatal air pollutants
